# Enhancing psychological well-being in college students: the mediating role of perceived social support and resilience in coping styles

**DOI:** 10.1186/s40359-024-01902-7

**Published:** 2024-07-15

**Authors:** Shihong Dong, Huaiju Ge, Wenyu Su, Weimin Guan, Xinquan Li, Yan Liu, Qing Yu, Yuantao Qi, Huiqing Zhang, Guifeng Ma

**Affiliations:** 1 School of Public Health, Shandong Second Medical University, No. 7166, Baotong West Street, Weicheng District, Weifang City, 261053 China; 2 Shandong Cancer Research Institute (Shandong Tumor Hospital), No.440, Jiyan Road, Huaiyin District, Jinan, 250117 China; 3 The First Affiliated Hospital of Shandong Second Medical University (Weifang People’s Hospital), No.151 Guangwen Street, Weicheng District, Weifang City, 261041 China

**Keywords:** College students, PC/NC, Depression, Perceived social support, Resilience

## Abstract

**Background:**

The prevalence of depression among college students is higher than that of the general population. Although a growing body of research suggests that depression in college students and their potential risk factors, few studies have focused on the correlation between depression and risk factors. This study aims to explore the mediating role of perceived social support and resilience in the relationship between trait coping styles and depression among college students.

**Methods:**

A total of 1262 college students completed questionnaires including the Trait Coping Styles Questionnaire (TCSQ), the Patient Health Questionnaire-9 (PHQ-9), the Perceived Social Support Scale (PSSS), and the Resilience Scale-14 (RS-14). Common method bias tests and spearman were conducted, then regressions and bootstrap tests were used to examine the mediating effects.

**Results:**

In college students, there was a negative correlation between perceived control PC and depression, with a significant direct predictive effect on depression (*β* = -0.067, *P* < 0.01); in contrast, negative control NC showed the opposite relationship (*β* = 0.057, *P* < 0.01). PC significantly positively predicted perceived social support (*β* = 0.575, *P* < 0.01) and psychological resilience (*β* = 1.363, *P* < 0.01); conversely, NC exerted a significant negative impact. Perceived social support could positively predict psychological resilience (*β* = 0.303, *P* < 0.01), and both factors had a significant negative predictive effect on depression. Additionally, Perceived social support and resilience played a significant mediating role in the relationship between trait coping styles and depression among college students, with three mediating paths: PC/NC → perceived social support → depression among college students (-0.049/0.033), PC/NC→ resilience → depression among college students (-0.122/-0.021), and PC/NC → perceived social support → resilience → depression among college students (-0.016/0.026).

**Conclusion:**

The results indicate that trait coping styles among college students not only directly predict lower depression but also indirectly influence them through perceived social support and resilience. This suggests that guiding students to confront and solve problems can alleviate their depression.

## Introduction

Depression is a complex mental disorder, characterized by cognitive, affective and psychosocial symptoms [1, 2]. It is projected that by 2030, depression will rank first globally in terms of years lived with disability [3, 4]. Depression is also one of the most common mental health issues among contemporary college students [5, 6]. Studies have shown that the detection rate of depression among Chinese college students ranges from 23–34% [7, 8]. Compared to non-student populations, college students have a higher prevalence of depression, and this rate seems to be increasing [9]. This vulnerable group of college students is in a unique developmental stage, facing pressures not only from life but also from the demands of academic coursework and complex interpersonal relationships, making the factors influencing depression among college students, particularly complex [9, 10].

Exploring the mechanisms by which influencing factors affect the occurrence of depression in college students is of significant importance for early prevention [11]. Research has demonstrated that trait coping style is one of the risk factors for depression among college students. Trait coping refers to the strategies individuals employ in challenging situations, categorized into positive coping and negative coping [12, 13]. Positive coping focuses on taking effective action and changing stressful situations, typically associated with problem-solving behaviors and regulation of positive emotions, which can help reduce the incidence of depression [14]. Conversely, negative coping is a passive approach centered around negative evaluations and emotional expression, often involving avoiding problems and social isolation, which is more likely to lead to the development of depression [14]. Research indicates that positive coping strategies are inversely correlated with depression, serving as protective factors against depression. Conversely, negative coping strategies are positively associated with depression, acting as risk factors for its onset [15].

Perceived social support refers to an individual’s subjective emotional state of feeling supported and understood by family, friends, and other sources [16, 17]. Prior studies have shown that perceived social support can directly impact an individual’s level of depression and also have indirect effects [18]. The data indicate that social support can significantly influence coping mechanisms, with groups having higher levels of social support tended to respond more actively and positively to stress from various sources [19]. Social support is considered an important mediating factor in determining the relationship between psychological stress and health, representing an emotional experience where individuals feel supported, respected, and understood [16]. The relationship between individuals’ coping strategies and depression may be influenced by the mediating role of perceived social support [20, 21]. In addition to this, resilience plays a role in all three.Resilience refers to the ability to adapt to stress and adversity, enhancing an individual’s psychological well-being [22]. Both coping styles and perceived social support significantly predict resilience positively [23]. For individuals with strong resilience, possessing a high level of adaptive capacity can mitigate the negative effects of stress on individuals, thereby enhancing their mental health.

In recent years, there has been a growing body of research on the prevalence of depression among college students. However, the rates of depression vary in different environments, and there is limited research on the mechanisms through which trait coping styles, perceived social support, and resilience impact depression. Therefore, this study aims to investigate the mechanisms through which positive coping styles(PC), negative coping styles(NC), perceived social support, and resilience influence depression among college students. Additionally, it seeks to analyze the mediating roles of perceived social support and resilience in this context. The goal is to provide insights into the reasons behind depression among college students under different coping strategies, aiding in timely psychological adjustment to promote the comprehensive development of the mental and physical well-being of college students.

The following assumptions were made:

### Hypothesis 1

PC has a significant negative predictive effect on depression among college students. NC has a significant positive predictive effect on depression among college students.

### Hypothesis 2

Perceived social support serves as a mediator between PC/NC and depression among college students.

### Hypothesis 3

Resilience mediates the relationship between PC/NC and depression among college students.

### Hypothesis 4

Perceived social support and psychological resilience mediate the relationship between PC/NC and depression among college students in a serial manner.

## Data and methods

### Data

This is a cross-sectional study that was conducted from January through February 2024. Using the Questionnaire Star network platform, we presented the questionnaire online, which was openly accessible to college students at a university in Shandong. The average time to complete the survey was 15 min. Participation was voluntary and students were informed about the purpose of the study. Confidentiality was assured and questionnaires were submitted anonymously. A total of 1267 enrolled college students participated in the questionnaire survey. After excluding invalid questionnaires, 1262 valid questionnaires were included, resulting in an effective rate of 99.57%.

### Methods

#### Trait coping style questionnaire

The Trait Coping Style Questionnaire (TCSQ) [24], developed by Qianjin Jiang, was utilized to assess the trait coping styles of college students. This questionnaire reflects the participants’ approaches to coping with situations, comprising a total of 20 items. It consists of two dimensions: negative coping style and positive coping style, each with 10 items. Using a 5-point Likert scale ranging from “definitely not” to “definitely yes,” scores were assigned from 1.00 to 5.00. The Cronbach’s α coefficient for negative coping style was 0.906 and for positive coping style was 0.786 in this study.

#### Depression scale

The Patient Health Questionnaire-9 (PHQ-9) [25] was used to assess depressive symptoms in the past two weeks. This scale consists of 9 items rated on a 4-point Likert scale ranging from “not at all” to “nearly every day,” with scores from 0 to 3. The total score ranges from 0 to 27, with higher scores indicating more severe depressive symptoms. The Cronbach’s α coefficient for this scale in the current study was 0.884.

#### Perceived Social Support Scale

The Perception Social Support Scale (PSSS) was compiled by James A.Blumenthal in 1987 and later translated and modified by Qianjin Jiang to form the Chinese version of the Zimetm Perception Social Support Scale (PSSS) [26, 27]. PSSS comprises 12 self-assessment items rated on a 7-point Likert scale. The scale includes three dimensions: family support (items 3, 4, 8, 11), friend support (items 6, 7, 9, 12), and other support (items 1, 2, 5, 10), with a total score ranging from 12 to 84. Scores of 12–36 indicate low support, 37–60 indicate moderate support, and 61–84 indicate high support. The Cronbach’s α for this scale in the current survey was 0.968.

#### Resilience scale

The Resilience Scale (RS-14) [28] Chinese version consists of 14 items, each rated on a 7-point Likert scale from “not at all” to “completely,” with scores ranging from 1 to 7. The total score ranges from 14 to 98, with higher scores indicating better resilience. The Cronbach’s α for this scale in the current study was 0.925.

### Statistical analysis

Data were organized and analyzed using SPSS 26.0 software. Confirmatory factor analysis was first conducted on the questionnaires. Descriptive analysis was then performed on the scores of each scale. Spearman was used to examine the relationships between trait coping styles, perceived social support, resilience, and depression. Mediation analysis was carried out using the SPSS PROCESS macro 3.4.1 software model 6 developed by Hayes, specifically designed for testing complex models. Model 6 was applied for two mediating variables, followed by the bias-corrected percentile Bootstrap method with 5000 resamples to estimate the 95% confidence interval of the mediation effect. A significant mediation effect was indicated if the 95% confidence interval (CI) did not include zero. A significance level of *P* < 0.05 was considered statistically significant.

## Results

### Examination of common method bias

Systematic errors in indicator data results caused by the same data collection method or measurement environment can typically be assessed through the Harman single-factor test on 55 items in the dataset to examine common method bias. The results indicated that there were 7 factors with eigenvalues greater than 1, and the variance explained by the first factor was 34.84%, which was below the critical threshold of 40%. Therefore, this study may not have a significant common method bias.

### Descriptive statistics and correlation analysis

The mean scores, standard deviations, and correlations of each variable are presented in Table 1. PC (*r*= -0.326, *P* < 0.01), resilience (*r*=-0.445, *P* < 0.01), and perceived social support (*r*=-0.405, *P* < 0.01) were negatively correlated with depression. PC (*r* = 0.336, *P* < 0.01) and resilience (*r* = 0.469, *P* < 0.01) were significantly positively correlated with perceived social support. PC was significantly positively correlated with resilience(*r* = 0.635, *P* < 0.01). NC was significantly positively correlated with depression(*r* = 0.322, *P* < 0.01) and PC(*r* = 0.146, *P* < 0.01). NC was significantly negatively correlated with perceived social support (*r*=-0.325, *P* < 0.01).

**Table 1:** Mean, standard deviation, and correlation analysis among variables

	Min	Max	M	SD	1	2	3	4	5
1. PC	10.00	50.00	35.95	6.01	1				
2. NC	10.00	50.00	29.57	8.22	0.146**	1			
3. Resilience	14.00	98.00	74.71	14.30	0.635**	-0.143**	1		
4. Perceived Social Support	12.00	83.00	66.75	12.91	0.336**	-0.325**	0.469**	1	
5. Depression	0	27.00	3.79	4.19	-0.326**	0.322**	-0.445**	-0.405**	1

Note: * indicates *P* < 0.05, ** indicates *P* < 0.01

### Analysis of chain mediation effects

**Fig. 1:**
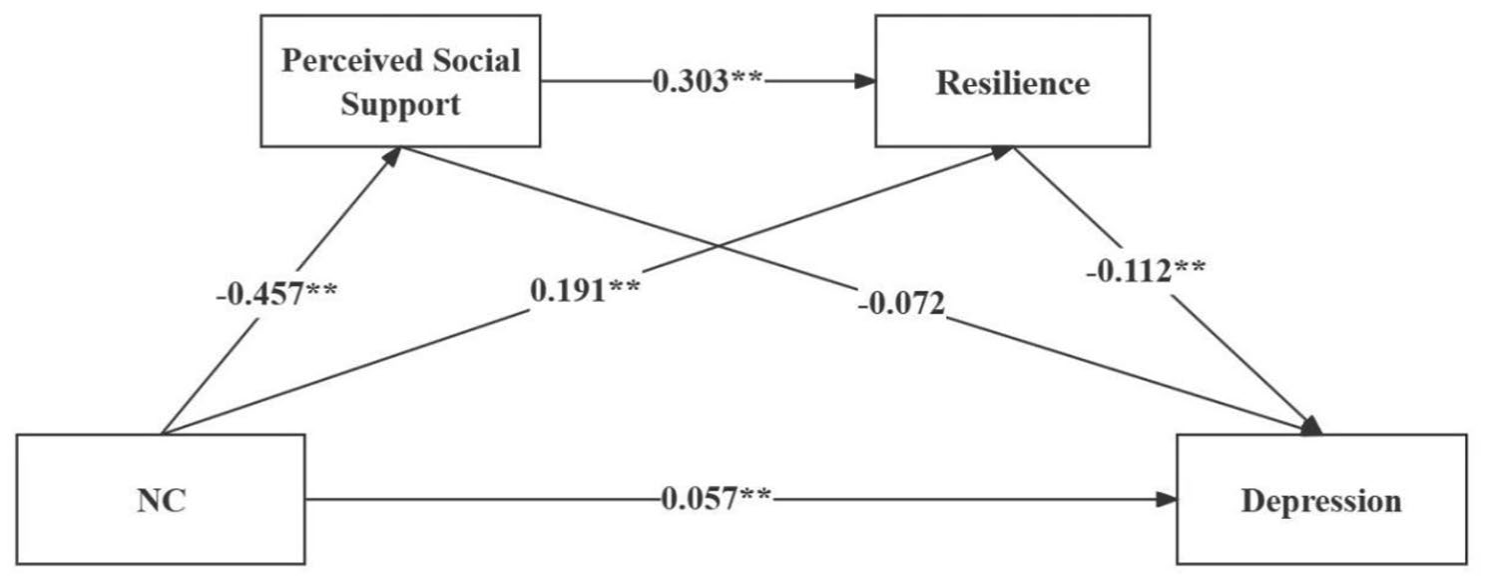
Chain mediation model of perceived social support and resilience between PC and depression. **p < 0.01

**Fig. 2:**
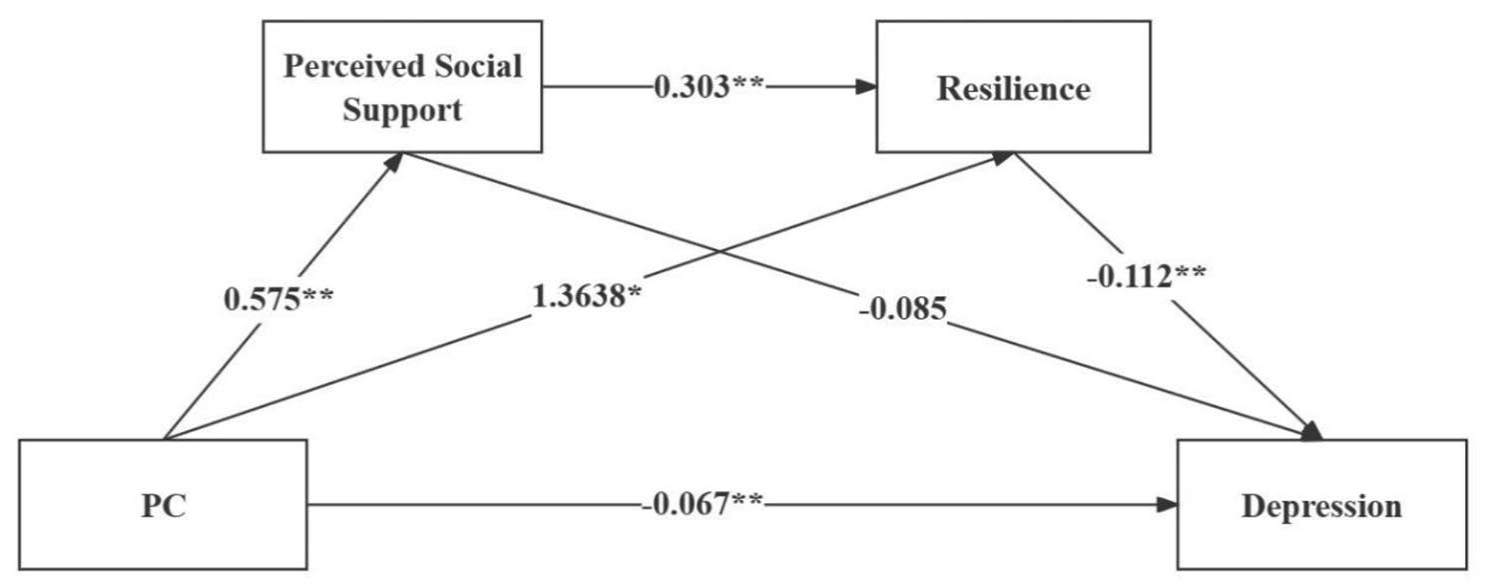
Chain mediation model of perceived social support and resilience between NC and depression. **p < 0.01

The chain mediation model was validated using SPSS PROCESS Model 6. Trait coping styles were considered as the independent variable, while depression among college students was treated as the dependent variable. Perceived social support and resilience were included as the mediating variables, culminating in the path model depicted in Figs. 1 and 2.

The results of the regression analysis, as shown in Table 2, indicated that PC could significantly predict perceived social support in a positive direction (*β* = 0.575, *P* < 0.01). Both PC (*β* = 1.363, *P* < 0.01) and perceived social support (*β* = 0.303, *P* < 0.01) had significant positive predictive effects on psychological resilience. When simultaneously predicting depression using PC, perceived social support, and psychological resilience, all three exhibited significant negative predictive effects (*β* = -0.067, *β* = -0.085, *β* = -0.090, *P* < 0.01). NC could significantly predict perceived social support in a negative direction (*β* = -0.457, *P* < 0.01). When NC (*β* = 0.191, *P* < 0.01) and perceived social support (*β* = 0.508, *P* < 0.01) jointly predict psychological resilience, they both had significant positive predictive effects. When simultaneously predicting depression using NC, perceived social support, and psychological resilience, NC (*β* = 0.057, *P* < 0.01) showed a significant positive predictive effect, while perceived social support (*β* = -0.072, *P* < 0.01) and psychological resilience (*β* = -0.112, *P* < 0.01) demonstrated significant negative predictive effects.

**Table 2:** Regression models of the mediation effects of social support and resilience

Outcome variable	Predictive variable	*R* ^2^	F	β	t	*P*
PS	PC/NC	0.072/0.085	97.168/116.496	0.575/-0.457	9.857/-10.793	< 0.01
R	PC/NC	0.487/0.193	598.170/150.839	1.363/0.191	27.365/4.150	< 0.01
	PS			0.303/0.508	13.061/17.344	< 0.01
D	PC/NC	0.292/0.298	173.026/178.034	-0.067/0.057	-3.083/4.497	< 0.01
	R			-0.085/-0.072	9.993/-8.107	< 0.01
	R			-0.090/-0.112	9.258/-14.556	< 0.01

Note: PC: positive coping, NC: negative coping, PS: Perceived Social Support Scale, R: Resilience Scale, D: Depression

Further employing the Bootstrap sampling method, with 5000 repetitions, the significance of the mediating effects and chain mediation effects between trait coping styles and depression among college students was examined. The results indicated that the direct effects of PC/NC on depression were significant, with direct impact values of -0.067/0.057 (26.38%/60.00%). Perceived social support and psychological resilience mediated the relationship between PC/NC and depression, with this mediation encompassing three pathways: the separate mediating effect of perceived social support, with effect values of -0.049 and 0.033 respectively; the separate mediating effect of resilience, with effect values of -0.122 and − 0.021 respectively; and the serial mediating effect from perceived social support to resilience, with effect values of -0.016, -0.021, and 0.026. The 95% confidence intervals for all pathways did not include 0, indicating significant indirect effects. Therefore, the total indirect effects were − 0.187 (73.62%) and 0.038 (40.00%), showing that PC had a weaker direct effect on depression compared to NC, but a stronger indirect effect. This was illustrated in Table 3.

**Table 3:** Examination of the mediating effects of perceived social support and resilience in the relationship between PC/NC and depression

			95%CI		
	Effect	SE	BootLLCI	BootULCI	Proportion of effect size
Total effects	-0.254	0.018	-0.289	-0.218	
Direct effects	-0.067	0.022	-0.109	-0.024	26.38%
Indirect effects	-0.187	0.029	-0.232	-0.145	73.62%
PC→PS→D	-0.049	0.015	-0.074	-0.029	19.29%
PC→R→D	-0.122	0.026	-0.161	-0.087	48.03%
PC→PS→R→D	-0.016	0.006	-0.025	-0.009	6.30%
Total effects	0.095	0.014	0.067	0.122	
Direct effects	0.057	0.013	0.032	0.082	60.00%
Indirect effects	0.038	0.01	0.017	0.058	40.00%
NC→PS→D	0.033	0.007	0.02	0.048	34.74%
NC→R→D	-0.021	0.007	-0.036	-0.007	-
NC→PS→R→D	0.026	0.004	0.019	0.034	27.37%

Note: PC: positive coping, NC: negative coping, PS: Perceived Social Support Scale, R: Resilience Scale, D: Depression

## Discussion

Previous research on the associations and specific pathways among depressive symptoms, trait coping styles, perceived social support, and resilience in college students has been limited. Therefore, this study utilized a chain mediation model to examine how trait coping styles, perceived social support, and resilience influence depressive symptoms in college students. The results indicate that perceived social support and resilience not only act as separate mediators between PC/NC and depression but also exhibit a chain mediation effect.

### **Mechanisms of the impact of** PC/NC **on depression in college students**

This study found that trait coping styles can significantly and negatively predict depressive symptoms in college students directly, consistent with previous research [29]. In recent years, amidst the backdrop of the pandemic, numerous studies have emerged domestically and internationally focusing on college students’ mental health from the perspective of crisis event coping [30]. These studies have predominantly concentrated on trait coping styles as a mediating variable in predicting the occurrence of depressive symptoms, with fewer studies examining the direct impact of trait coping styles on depressive symptoms. College students, being in a unique developmental stage, face challenges from various aspects and bear the pressures of academic coursework, interpersonal relationships, and future employment. Research indicates that trait coping styles are a key factor influencing mental health [31]. Implementing healthy coping techniques and interventions can help individuals overcome negative emotions caused by stress, which is an adaptive coping mechanism that assists college students in facing stress and enhancing problem-solving abilities, thus preventing or reducing the occurrence of depression. Conversely, adopting passive or avoidant coping strategies, leading to inadequate resolution of stress events, can increase psychological stress [14], thereby exerting a negative impact on the mental health of college students [32]. Therefore, trait coping styles play a negative predictive role in depressive symptoms among college students. PC was a positive predictor of depression and NC was a negative predictor of depression. This is consistent with previous studies [24, 29].

### Separate mediating effects of perceived social support and resilience

After introducing perceived social support and resilience as two mediating variables, the predictive effect of PC/NC on depressive symptoms in college students remained significant. The results show that PC can positively predict perceived social support, and NC is the opposite, consistent with previous research [33]. Trait coping styles are an important predictive factor in altering college students’ perceptions of social support and the occurrence of depression. Individuals who adopt negative coping styles tend to perceive relatively less external support. Some argue that social support plays a reverse predictive role in trait coping styles; the more social support college students receive and feel, the more likely they are to actively adopt positive coping strategies to alleviate stress, potentially due to variations in study subjects and time [34]. In this pathway, perceived social support can significantly and negatively predict depressive symptoms, aligning with previous research findings [35]. Perceived social support is considered a crucial mediating factor influencing mental health, referring to an individual’s ability to perceive support and understanding from family, friends, and others. College students with lower levels of perceived social support often feel neglected and undervalued, leading to negative evaluations and self-doubt, making them more susceptible to depression. PC/NC and perceived social support can interact and influence the occurrence of depressive symptoms in college students [16].

Research indicates that PC can significantly and positively predict resilience, with an indirect effect value of 48.03%.In this pathway, the mediating effect of resilience is more pronounced, consistent with previous studies [36]. There is a close connection between resilience and coping styles; college students who adopt positive coping strategies often exhibit stronger psychological resilience, being more willing to confront issues and seek help from others to solve problems. When facing pressures such as academic challenges, they approach them with a positive mindset, overcoming adversity [37]. It is believed that adopting positive coping strategies to address problems can enhance college students’ levels of psychological resilience [10, 38]. Resilience can significantly and negatively predict depressive symptoms. depressive symptoms, College students with higher levels of resilience tend to define the severity of events less severely when stress events occur, resulting in lower psychological burdens and reduced likelihood of experiencing depressive symptoms [10]. Additionally, when facing setbacks or stress, individuals who adopt positive coping strategies actively utilize internal and external protective factors to combat current difficulties and pressures, and employ effective emotional control to mitigate the impact, thereby enhancing their levels of psychological resilience and reducing the occurrence of depression.

### Chain mediation effect of perceived social support and psychological resilience

This study elucidates that PC/NC perceived social support, and psychological resilience are independent factors influencing depressive symptoms in college students, with perceived social support and psychological resilience playing a mediating role between coping styles and depressive symptoms. The share of total indirect effect values is 73.62% and 40.00%, respectively, with the third chain path accounting for 6.30% and 27.37% of the total effect ratio, respectively. This confirms the existence of this chain mediation effect, although the chain mediation effect is not as pronounced as the individual mediation effects. Positive coping styles not only directly negatively predict depressive symptoms in college students but also exert an indirect influence on depressive symptoms through perceived social support and psychological resilience. Likewise, negative coping styles not only directly positively predict depressive symptoms in college students but also have an indirect impact on depressive symptoms through perceived social support and psychological resilience, thus demonstrating the value and significance of these two mediating variables in reducing the occurrence of depressive symptoms in college students.

Initially, adopting positive coping styles and being able to perceive social support are crucial factors influencing psychological resilience in college students. There exists a relatively stable systemic relationship between students’ social support and psychological resilience, confirming that social support can enhance individuals’ levels of psychological resilience [16]. Furthermore, coping styles can affect the occurrence of depressive symptoms from both internal and external perspectives. This is because the social support perceived by college students includes not only tangible social support resources but also their subjective perception of social support, with these two factors constituting external and internal protective factors of psychological resilience [39]. Positive coping and effective adaptation can enhance college students’ perception of social support, enabling them to mobilize personal, familial, and societal protective factors better when facing various life challenges, thereby mitigating or eliminating difficulties and suppressing the onset of depressive symptoms, whereas negative coping styles yield the opposite effect. The chain mediation proposed in this study integrates the research on perceived social support, psychological resilience, and depressive symptoms in college students, facilitating a more comprehensive understanding of the internal mechanisms through which coping styles influence depressive symptoms in college students. This holds significance in advocating for a proactive attitude in college students to confront and resolve difficulties and in increasing attention to the mental health of college students.

### Limitations, strengths and future research

The findings of this study hold theoretical value and practical implications, offering a reference basis for improving the mental health of college students. However, there are certain limitations to consider. Firstly, the survey in this study was conducted through self-reporting, which may introduce certain biases. Future research could explore data collection through various methods. Secondly, this study employed a cross-sectional design to investigate the impact of trait coping styles, on depression among college students and its potential mechanisms. However, this research approach does not allow for causal inferences between variables, and further validation of the study’s conclusions could be achieved through longitudinal or experimental research.

## Conclusion

In summary, this study aims to improve the mental health of college students by examining how their coping styles, along with their perceived social support and psychological resilience, affect depressive symptoms. The research analyzes the connections between these factors and suggests that positive coping styles may help prevent depression. However, the study has its limitations and future research should use long-term experiments to better understand these relationships. Since depression in college students can be influenced by many factors, future studies should also consider additional variables and use a mix of experimental and longitudinal approaches to more clearly understand how to reduce depression in this group.

## Data Availability

The datasets used and analysed during the current study are available from the corresponding author upon reasonable request.

## Abbreviations

PHQ-9: Patient Health Questionnaire-9
PSSS: Perceived Social Support Scale
TCSQ: Trait Coping Style Questionnaire
PC: Positive coping styles
NC: Negative coping styles
RS-14: Resilience Scale
